# Supplementary material for: Deep Learning Approach Predicts Longitudinal Retinal Nerve Fiber Layer Thickness Changes
Source: Bioengineering (Basel). 2025 Jan 31;12(2):139. doi: 10.3390/bioengineering12020139 (PMC11851649; doi:10.3390/bioengineering12020139)
Supplement: Supplementary file 1 [file bioengineering-12-00139-s001.zip › bioengineering-3417475-supplementary.pdf]

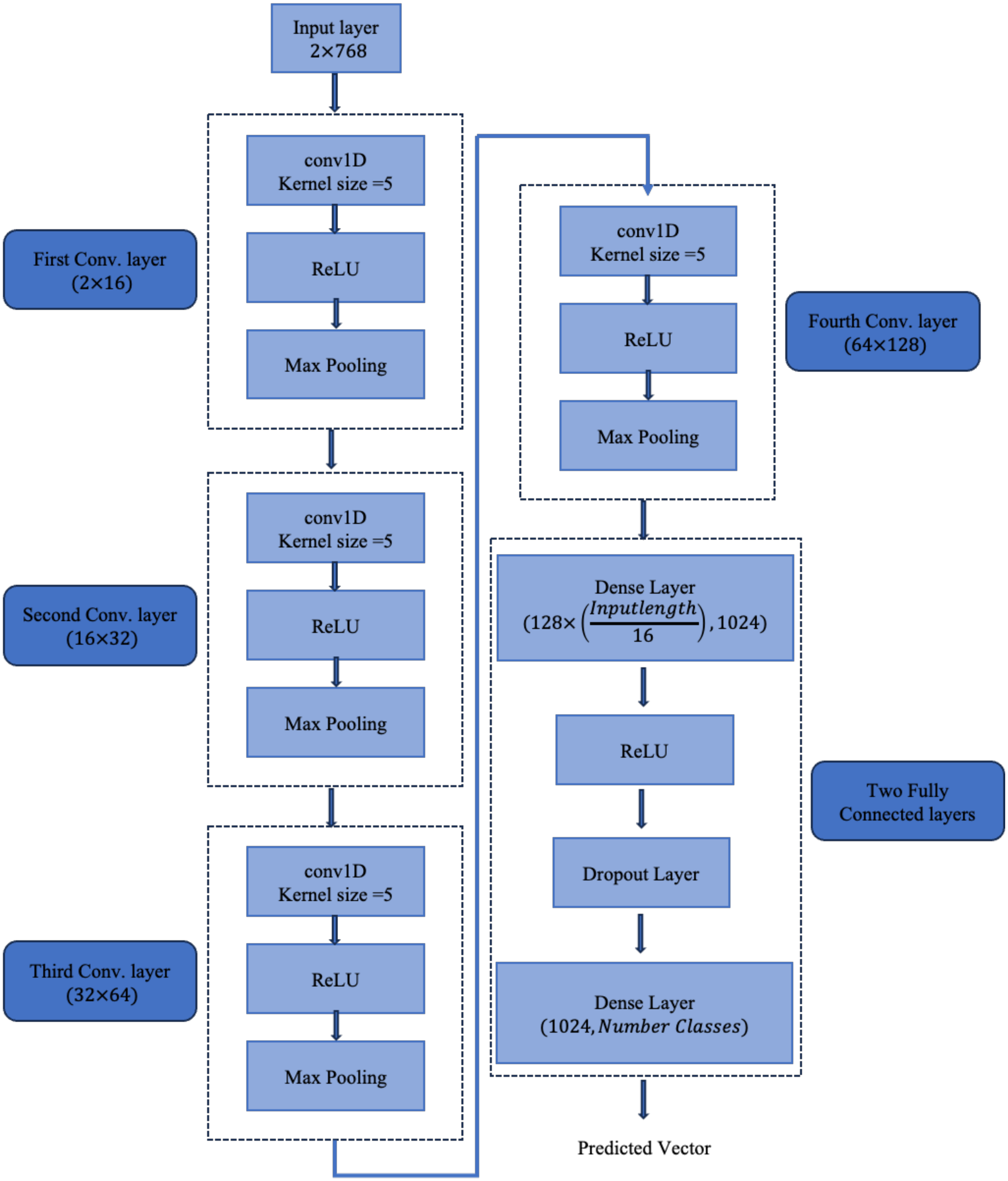

Figure S1: Schematic of the one-dimensional convolutional neural network (1D-CNN) used to predict future RNFL thickness.
